# Family-Based Bivariate Association Tests for Quantitative Traits

**DOI:** 10.1371/journal.pone.0008133

**Published:** 2009-12-02

**Authors:** Lei Zhang, Aaron J. Bonham, Jian Li, Yu-Fang Pei, Jie Chen, Christopher J. Papasian, Hong-Wen Deng

**Affiliations:** 1 Key Laboratory of Biomedical Information Engineering, Ministry of Education and Institute of Molecular Genetics, School of Life Science and Technology, Xi'an Jiaotong University, Xi'an, Shaanxi, People's Republic of China; 2 School of Medicine, University of Missouri-Kansas City, Kansas City, Missouri, United States of America; 3 Department of Mathematics and Statistics, University of Missouri-Kansas City, Kansas City, Missouri, United States of America; 4 Center of System Biomedical Sciences, Shanghai University of Science and Technology, Shanghai, People's Republic of China; 5 College of Life Sciences and Engineering, Beijing Jiao Tong University, Beijing, People's Republic of China; University of Montreal, Canada

## Abstract

The availability of a large number of dense SNPs, high-throughput genotyping and computation methods promotes the application of family-based association tests. While most of the current family-based analyses focus only on individual traits, joint analyses of correlated traits can extract more information and potentially improve the statistical power. However, current TDT-based methods are low-powered. Here, we develop a method for tests of association for bivariate quantitative traits in families. In particular, we correct for population stratification by the use of an integration of principal component analysis and TDT. A score test statistic in the variance-components model is proposed. Extensive simulation studies indicate that the proposed method not only outperforms approaches limited to individual traits when pleiotropic effect is present, but also surpasses the power of two popular bivariate association tests termed FBAT-GEE and FBAT-PC, respectively, while correcting for population stratification. When applied to the GAW16 datasets, the proposed method successfully identifies at the genome-wide level the two SNPs that present pleiotropic effects to HDL and TG traits.

## Introduction

Recent technological advances in genotyping along with the capacity to detect increasingly large numbers of single nucleotide polymorphisms (SNPs) have created great demand for developing new strategies to identify genes that underlie phenotypic variation. The availability of high-throughput SNP genotype data is prompting the development of genetic association analyses, including family based association tests (FBAT).

For family data sets, such as the Framingham heart study [1], multiple phenotypes are usually recorded. While most of the current analyses focus only on traits individually, explicitly modeling genetic and environmental correlations among traits can theoretically extract more information and consequently provide a greater power of test. In linkage studies, it has been shown that joint analyses of two correlated traits may substantially improve power for localizing genes that jointly influence complex traits, and for evaluating their effects [2]–[7]. In association studies, however, only a limited few methods are available [8]–[10]. Therein, Lange et al. [10] proposed a multivariate generalized estimating equations (GEEs) based method, termed FBAT-GEE. The method FBAT-GEE makes no assumptions on phenotypic distributions and thus can be applied to phenotypes with arbitrary distributions. For quantitative traits, Lange et al. [9] also proposed a generalized principal component analysis (PCA), termed FBAT-PC, which is more powerful than FBAT-GEE.

Both the methods FBAT-GEE and FBAT-PC possess the property of protection against population stratification by a transmission disequilibrium test (TDT)-like strategy. Despite its potential merit, this property comes at the cost of a substantial loss of statistical power by the fact that genotypes at each single marker need to be decomposed in order to correct for population stratification and test association simultaneously. The loss of power may become problematic in the context of genomewide association studies (GWAS) where it is critical to achieve a genomewide significance level in order to judge a positive finding.

Alternatively, the issue of population stratification can be handled at the population level by studying population structures from genotype data of multiple markers [11]–[17]. Among these approaches, Principal component analysis based methods [12], [14], [16], [17] summarize individual genetic background information. PCA-based methods are proven to be both computationally fast and statistically effective. The extensions of PCA to familial data are also proposed by Zhu et al. [14] and by us previously [18]. Thus, with the availability of large numbers of genotyped markers, a more flexible scheme that would enhance the feasibility of applying FBAT would be to correct for population stratification from multiple markers rather than from any single marker.

In this study, under the framework of the variance-components (VCs) model [19], [20], we develop a method for tests of association by joint analysis of two correlated quantitative traits in families. Specifically, Individual genotype scores and phenotypes are adjusted by the use of the principal component analysis to guide against potential population stratification. A score test is proposed with the residual of genotypes and of phenotypes. Statistical properties of the proposed method are investigated through extensive simulations under a variety of conditions, and its performance is compared with existing both univariate and bivariate methods.

## Methods

### Multivariate Variance-Components Pedigree Model

We describe the problem in the variance-components (VCs) [8], [19], [20] framework which is a powerful tool for modeling phenotypic variation for continuous traits in families. We describe the model in the context of multivariate phenotypes, although we consider only bivariate situation in our analysis.

Assume that there are *N* nuclear families with *n_i_* individuals in the *i*-th family (*i* = 1, …, *N*). Let **y** *_ij_* = (*y_ij_*_1_, …, *y_ij_*_m_)′ be the vector of *m* (*m* = 2 for bivariate) phenotypes for individual *j* (*j* = 1, …, *n_i_*) in family *i*. Further, let **Y** *_i_* = (**y** *_i_*_1_′, …, **y** *_in_i__*′)′ be the vector of phenotypes for all members in family *i*. Under the additive mode of inheritance, the genotype score *g_ij_* is defined as 0, 1 and 2 for genotypes “11”, “12” and “22”, respectively. In the variance-components model, genetic components contributing to phenotypes are decomposed into the fixed effects, e.g., the effects at the specified locus, and the random effects, e.g., the effects of unknown polygenes. Specifically, the phenotype vector **y** *_ij_* can be described by the following multivariate variance-components model
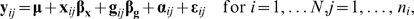
(1)where 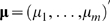 denotes the vector of grand means for the *m* phenotypes; **x** *_ij_* is a *m*×*t* design matrix for *t* covariates, e.g., age, sex, and known environment factors, to the *m* phenotypes, and **β_x_** is the vector of corresponding covariate effects; **g** *_ij_* is a *m*×*m* design matrix for genotype scores with the *m* principal diagonal elements being *g_ij_* and the other elements being 0, and **β_g_** the corresponding additive major gene effects. At last, **α** *_ij_* and **ε** *_ij_* are vectors of length *m* denoting, respectively, the additive polygenic effects and the residual effects.

Here, the covariate effects 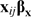 and the major gene effects 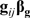 are modeled as fixed, whereas the polygene effects 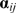 and the residuals 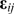 are modeled as random. Let 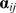 and 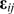 follow multivariate normal distributions
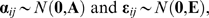
where **A** and **E** are the *m*×*m* variance-covariance matrices accounting for polygenic (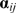) and environmental (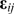) variation among the traits, respectively, so that
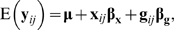
(2)The covariance matrix of **y** *_ij_*, 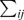, has elements
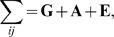
where **G**, **A**, and **E** are the *m*×*m* variance-covariance matrices accounting for major gene (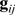), polygenic (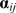) and environmental (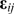) variations, respectively.

The phenotype vector for the family *i*, **Y** *_i_*, will then follow a multivariate normal distribution with the mean vector
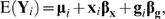
(3)and the covariance matrix
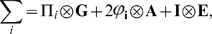
(4)where 
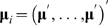
 is the mean vector with length *mn_i_* for the family *i*; 
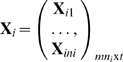
 is the design matrix for covariates, and 
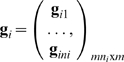
 is the design matrix for genotypes at the tested locus; 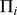 is the *n_i_*×*n_i_* identity-by-descent (IBD) matrix at the tested locus (estimated from the genotype data) and 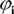 is the *n_i_*×*n_i_* kinship coefficient matrix (estimated from the relationships among individuals), both of which can be calculated from pedigree structures and available genotype data. Finally, **I** is the *n_i_*×*n_i_* identity matrix and 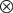 is the Kronecker-product operator for matrices.

### Correcting for Population Stratification

When the issue of population stratification exists, the model described above may not provide a valid test. We previously proposed to extend the principal component analysis to include familial data [18]. The method is briefly outlined as follows: founders in each family are selected to form an unrelated sample on which principal component analysis is performed with available genotype data. The calculated principal components are used to estimate these founders' genetic background information and to adjust their genotype scores and phenotypes, as described by Price et al. [12]. Principal components for non-founders in each family are inferred according to those for their founder relatives through a TDT strategy. The inferred principal components are then used to adjust non-founders' genotypes and phenotypes. The approach is also extended to the scenarios where parental information is missing. Denote the adjusted genotypes and phenotypes with an asterisk (*), and we rewrite the equation (3) as
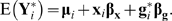
(5)


### Tests of Association

With the assumptions of independent families and of within-family multivariate normality distributed phenotypes, the likelihood function of the adjusted genotype and phenotype data is written as
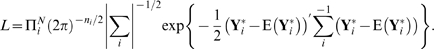
Evidence of association is assessed by a statistical hypothesis testing of the null hypothesis *H*_0_: 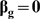 (no association) versus the alternative hypothesis *H*_1_: 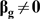 (evidence of association). Generally, the hypothesis can be tested by a likelihood ratio test (LRT) where for each marker the maximal likelihoods under the null and alternative hypotheses are estimated respectively. However, the LRT is rather computationally intensive when large numbers of markers are involved, making it prohibitive for large-scale scans. Here we propose a multivariate score test as the extension of that proposed by Chen & Abecasis [21]. The set of parameter in the likelihood function is 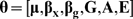. We first fit the model under the null hypothesis (without 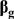), from which we obtain the maximal likelihood estimates (MLE) of 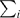 and 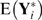, denoted by 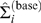 and 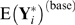, respectively, for family *i*. Under the null hypothesis, the score vector with respect to **β_g_** is defined as
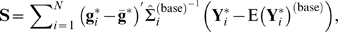
and the corresponding variance-covariance matrix is
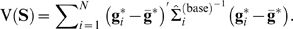
The score test statistic is then defined as
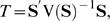
which asymptotically follows a 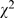 distribution with degree of freedoms (df) being the rank of 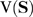, which is equal to *m* unless there are linear dependences between the phenotypes. The statistic *T* is valid regardless of the presence of population stratification.

### Data Simulations

Statistical properties and performances of the proposed method were investigated via extensive simulation studies. For genotype data, we simulated 998 SNP markers, with the allele frequency for each marker being drawn from the Uniform distribution U(0.1, 0.9). We also simulated two additional SNPs, both with MAF 0.3, as the causal and the test SNPs, respectively. Two hundred nuclear families were simulated by sampling parental genotypes according to allele frequencies, and then by randomly selecting two parents to produce children. Unless otherwise specified, the number of children per family was drawn from a Poisson distribution with mean 2.

Two quantitative traits were simulated by the equations (3) and (4). To each trait, the causal SNP was assumed to explain a specific proportion of phenotypic variation, which was set to 2% by default, and the background polygenic effects were assumed to explain additional 40% of phenotypic variation. The polygenic (*ρ_a_*) and environmental (*ρ_e_*) correlations between the traits were set to 0.4 unless otherwise specified.

When needed, population stratification was generated by mixing equal numbers of families from two discrete populations A and B. Marker allele frequencies in the two populations were generated using the Balding-Nichols model [22]. Specifically, for each SNP, an ancestry allele frequency *p* was drawn from the Uniform distribution U(0.1, 0.9). The allele frequencies for populations A and B were then drawn from a Beta distribution with parameters *p*(1−*F_ST_*)/*F_ST_* and (1−*p*)(1−*F_ST_*)/*F_ST_*, where *F_ST_* is the measure of genetic distance between the two populations. We set *F_ST_* to 0.05 to simulate moderate population stratification. The two populations were generated separately with different phenotypic means (*μ_A_* and *μ_B_*) and different causal and test SNP MAFs (*p_A_* = 0.2 and *p_B_* = 0.4 for both causal and test SNPs). The values of *μ_A_* and *μ_B_* were selected such that 20% of the phenotypic variance of each trait in the combined population was explained by stratification.

We evaluated the statistical properties, including type I error rates and power, of the proposed method. In all the simulations, the null hypothesis was produced by setting the LD measure *r* ^2^ between the causal and the test sites to 0.0, whereas the alternative hypothesis was produced by setting a certain value of *r* ^2^ between the two sites. Unless otherwise specified, the value of *r* ^2^ under the alternative was set to 1.0 to produce a perfect association between the two sites.

We also studied the effects of various influential factors, including locus effect, correlation coefficient between traits, the level of LD, and family structure, on the performance of the proposed method. For comparison, we also included extensive popular univariate and bivariate methods into analysis. For univariate method, we selected the commonly used method QTDT proposed by Abecasis et al. [23], and the univariate score test proposed by us previously [18]. For bivariate analyses, we selected two popular methods: FBAT-GEE [10] and FBAT-PC [9], which are implemented in the programs FBAT [24] and PBAT [25], respectively. We denote the proposed test and the other methods as T, UT, QTDT, FBAT and PBAT, respectively, throughout the study.

### GAW16 Datasets

As an application, we analyzed the Genetic Analysis Workshop 16 (GAW16) Problem 3 simulated data sets with the proposed method. The access and analyses of the GAW16 simulated data sets have been approved by the Institutional Review Board (IRB) of the University of Missouri-Kansas City (UMKC). The GAW16 data sets consist of 6476 subjects from the Framingham Heart Study (FHS), where each subject has real genotypes at approximately 550,000 (549,872) SNPs and simulated phenotypes.

Subjects are distributed among three generations and singletons. After dividing large families into smaller nuclear families and applying some quality controls to the data (for example, as the proposed test cannot analyze half-sibs, we deleted half-sibs from the data), we finally identified 5456 family members from a total of 1815 nuclear families.

A total of four correlated quantitative traits, termed HDL, LDL, TG, CHOL, respectively, are simulated in order to mimic the lipid pathway underlying the development of cardiovascular disease [26]. We focused on the traits HDL and TG. Genetic components underlying each of both traits consist of several SNPs with major effects and 1,000 SNPs with polygenic effects. Two major SNPs (*rs3200218* and *rs8192719*) present pleiotropic effects to both traits in the simulation. Phenotype data are simulated at three pseudo-visits with 10 years apart to mimic the context of longitudinal study, and at each visit, 200 simulated data sets are replicated. The dataset from the first replicate of the first visit was analyzed as suggested by the workshop. Both phenotypes were adjusted by age and sex.

## Results

### Type I Error Rates

We first estimate type I error rates under a variety of polygenic (*ρ_a_*) and environmental (*ρ_e_*) correlations in homogeneous population setting, as listed in Table 1. Two modes of linkage are considered: 1) the marker is tightly linked to but not associated with the QTL (Linkage); and 2) the marker is neither linked to nor associated with the QTL (No linkage). It is obvious that all methods have correct type I error rates that are close to the target levels, regardless of the existence of linkage.

**Table 1:** Type I error rates at various levels of residual correlations under homogeneous population.

	*ρ_a_*																
	−0.8			−0.4			0.0			0.4			0.8				
*ρ_e_*	T	FBAT	PBAT	T	FBAT	PBAT	T	FBAT	PBAT	T	FBAT	PBAT	T	FBAT	PBAT	QTDT	UT
Linkage^a^																	
−0.8	4.9	4.6	5.1	3.8	4.5	5.3	5.0	5.8	5.1	4.7	5.7	5.3	4.1	4.6	3.8	4.9	5.5
−0.4	7.4	5.8	5.2	5.2	4.6	5.2	5.1	5.7	4.4	5.0	4.5	4.9	5.2	6.0	4.8		
0.0	5.3	4.5	5.3	4.7	5.8	6.8	5.3	3.9	4.3	4.7	6.6	4.0	5.8	5.7	5.2		
0.4	5.3	4.8	4.2	5.3	4.5	5.1	5.1	3.7	4.6	5.0	5.2	5.8	4.3	4.6	5.5		
0.8	4.7	4.6	5.3	4.3	4.2	4.9	4.8	4.8	5.4	4.8	5.7	5.3	3.9	4.9	3.9		
No Linkage^b^																	
−0.8	4.7	4.5	3.8	5.7	4.9	5.4	4.6	4.6	4.2	5.8	4.9	4.2	5.9	4.3	4.9	5.2	4.0
−0.4	5.1	3.9	4.5	5.7	5.1	5.4	5.4	5.5	5.2	4.1	6.5	4.1	4.3	4.8	5.3		
0.0	5.5	4.8	3.9	4.4	4.2	5.1	3.8	6.5	5.8	4.0	6.0	5.1	4.3	5.2	4.1		
0.4	5.2	5.4	3.7	4.6	4.9	4.2	4.9	6.1	3.8	4.1	4.6	4.9	5.6	5.4	4.6		
0.8	5.2	6.2	5.2	6.4	4.1	4.4	4.4	5.0	4.8	4.3	5.4	6.0	4.2	4.5	4.6		

Two hundred nuclear families were simulated, with the number of children per family being drawn from a Beta distribution with mean 2. Type I error rate was estimated at nominal level 5% on 1,000 replicates, with various levels of polygenic correlation (*ρ_a_*) and environmental correlation (*ρ_e_*).

a the test site was linked to but not associated with the causal site.

b the test site was neither linked to nor associated with the causal site.

Abbreviations: T, the proposed bivariate method; FBAT, the method FBAT-GEE [10] implemented in the software FBAT [24]; PBAT, the method FBAT-PC [9] implemented in the software PBAT [25]; QTDT, the method proposed by Abecasis et al. [23] and implemented in the software QTDT; UT, the univariate test in our previous study [18].

Table 2 lists the type I error rates when families from two populations are admixed. All methods again have correct error rates, implying their ability to protect against population stratification.

**Table 2:** Type I error rates at various levels of residual correlations under admixed population.

	*ρ_a_*																
	−0.8			−0.4			0.0			0.4			0.8				
*ρ_e_*	T	FBAT	PBAT	T	FBAT	PBAT	T	FBAT	PBAT	T	FBAT	PBAT	T	FBAT	PBAT	QTDT	UT
Linkage^a^																	
−0.8	2.0	4.5	4.5	3.5	5.7	4.0	3.2	4.0	4.3	3.8	5.4	5.5	4.3	5.7	5.4	4.6	4.4
−0.4	3.5	5.4	5.7	3.3	4.1	5.1	4.1	5.2	5.8	2.8	3.9	5.7	3.8	4.1	4.0		
0.0	4.3	4.2	3.7	2.9	4.5	4.3	3.8	4.2	5.1	5.5	4.7	5.1	4.0	5.0	5.1		
0.4	2.8	4.0	4.9	2.7	4.4	4.5	3.0	5.3	3.8	2.2	3.9	4.3	3.2	4.9	3.7		
0.8	2.9	5.8	5.8	3.2	4.9	3.2	2.8	4.9	4.8	2.6	4.0	5.8	3.6	6.0	4.8		
No Linkage^b^																	
−0.8	1.9	5.2	4.2	2.6	4.9	5.7	3.6	5.1	4.9	2.9	4.8	4.0	4.1	4.6	6.0	5.0	4.5
−0.4	3.7	4.9	5.0	4.2	4.4	5.2	3.0	6.0	5.8	3.4	5.4	5.4	4.6	5.3	5.3		
0.0	2.8	4.6	4.4	3.1	5.4	5.9	3.4	5.8	5.0	3.8	6.0	5.5	4.5	4.8	5.0		
0.4	3.9	5.4	5.3	2.8	4.8	4.6	4.1	5.4	5.5	4.0	4.9	4.8	4.7	4.5	4.7		
0.8	3.7	5.1	5.9	4.4	5.1	5.8	4.1	4.8	5.3	3.9	6.5	5.7	3.9	4.2	4.7		

Two hundred nuclear families were simulated by admixing 100 from two populations A and B. See Table 1 legend for simulation and abbreviation details.

We also estimate the type I error rates when parents in each family are missing, as presented in table 3. In the table, the number of children per family varies from 2 to 4, with the total number of children being fixed at 480. Again, all investigated methods have correct type I error rates regardless of the presence of linkage or population stratification, indicating the proposed method as well as the others is applicable to studies with missing parental information.

**Table 3:** Type I error rates when parents are missing.

	No. of children per family														
	2					3					4				
	T	FBAT	PBAT	QTDT	UT	T	FBAT	PBAT	QTDT	UT	T	FBAT	PBAT	QTDT	UT
Homogeneous															
Linkage	6.3	4.7	5.9	4.5	4.7	3.3	5.1	3.6	5.2	4.9	5.3	4.7	5.0	5.2	4.2
No Linkage	5.0	3.3	4.3	4.7	3.6	4.9	4.1	4.6	4.9	4.7	5.9	4.7	5.0	4.8	5.3
Admixture															
Linkage	3.9	4.7	5.2	5.2	3.5	4.6	5.3	5.0	4.8	3.9	4.2	4.3	5.1	4.1	4.5
No Linkage	4.0	4.5	4.6	4.7	3.8	4.7	5.9	6.1	5.8	4.7	5.3	4.5	5.2	5.1	4.8

The number of children per family varied from 2 to 4, and the number of families varied accordingly with the constraint that the total number of children was fixed at 480. Both polygenic and environmental correlations were set to 0.4. See Table 1 legend for simulation and abbreviation details.

### Power Estimates

Powers of various methods affected by *ρ_a_* and *ρ_e_* are listed in table 4. For all the three bivariate approaches, power increases as residual correlations (*ρ_a_* and/or *ρ_e_*) decrease from +1.0 to −1.0. For example, under homogenous population, the power of the proposed method is 87.6% when both *ρ_a_* and *ρ_e_* are +0.8, and increases to 100.0% when both correlations decrease to −0.8. In additional simulations where the major gene correlation between the traits is constrained to −1.0 rather than +1.0, we observe an opposite trend in power change; power increases as *ρ_a_* and/or *ρ_e_* increase from −1.0 to +1.0 (data not shown). Therefore, our simulation results indicate that the power of the bivariate approaches increases when the effects of the major-gene and those of the residuals (polygenic and environmental) act in increasingly dissimilar directions, which coincides with previous studies in the literature of linkage analyses [3].

**Table 4:** Power estimates at various levels of residual correlations.

	*ρ_a_*																
	−0.8			−0.4			0.0			0.4			0.8				
*ρ_e_*	T	FBAT	PBAT	T	FBAT	PBAT	T	FBAT	PBAT	T	FBAT	PBAT	T	FBAT	PBAT	QTDT	UT
Homogeneous																	
−0.8	100.0	100.0	100.0	100.0	99.3	99.7	100.0	95.9	98.3	100.0	87.5	94.5	100.0	80.0	88.7	61.3	93.7
−0.4	100.0	97.3	99.5	100.0	90.9	96.1	100.0	83.8	92.0	99.8	74.9	85.7	99.3	71.5	82.4		
0.0	100.0	89.2	93.2	100.0	80.5	89.6	99.4	75.2	84.6	99.2	65.4	77.1	98.4	59.7	70.5		
0.4	100.0	79.0	88.4	99.4	70.6	79.7	98.0	62.8	76.5	97.4	60.3	71.8	96.1	53.7	65.8		
0.8	99.7	66.6	76.9	98.2	61.8	73.6	96.0	58.2	65.5	95.6	55.1	64.1	90.5	48.8	58.0		
Admixture																	
−0.8	100.0	85.8	94.5	99.7	76.0	83.1	99.9	71.2	80.3	100.0	64.5	74.6	99.8	57.7	66.8	55.1	91.4
−0.4	99.8	75.1	82.4	100.0	69.8	80.3	99.3	61.0	74.8	99.3	56.3	63.1	98.4	49.7	57.2		
0.0	99.9	64.0	78.3	99.3	57.9	64.7	98.0	53.3	57.6	97.5	50.9	54.2	94.9	44.7	50.3		
0.4	98.4	55.7	64.7	97.6	50.4	58.6	96.3	49.1	53.4	93.4	44.2	47.7	92.0	40.4	44.5		
0.8	95.6	48.8	55.5	94.0	45.5	52.2	92.3	40.4	48.0	91.3	35.7	42.5	87.6	37.4	39.6		

Power was estimated at the nominal level of 5% based on 1,000 replicates. The value of *r* ^2^ between test and causal sites was set to 1.0, and the recombination rate between the sites was set to 0.01. See Table 1 and 2 legends for simulation and abbreviation details.

Among the three bivariate methods, the proposed one has the highest power under all the parameter settings. The power improvement is remarkably large. For example, when both *ρ_a_* and *ρ_e_* are +0.8 under homogeneous population, the power for the proposed method is 90.5%, whereas it is only 48.8% for FBAT and 58.0% for PBAT. For the other two methods, PBAT has a higher power than FBAT.

When comparing the bivariate and univariate approaches, the proposed method has higher power than UT under most conditions, and than QTDT under all the conditions. UT has higher power than QTDT, consistent with our previous study [18]. FBAT and PBAT have higher power than QTDT unless the traits are highly positively correlated.

Power when parental information is missing is presented in table 5. The trends in power changes are similar with those when parental information is available. The proposed method again has the highest power, and the bivariate tests have higher power over univariate tests in most situations.

**Table 5:** Power estimates at various levels of residual correlations when parents are missing.

	*ρ_a_*																
	−0.8			−0.4			0.0			0.4			0.8				
*ρ_e_*	T	FBAT	PBAT	T	FBAT	PBAT	T	FBAT	PBAT	T	FBAT	PBAT	T	FBAT	PBAT	QTDT	UT
Homogeneous																	
−0.8	99.0	100.0	100.0	99.2	96.7	99.2	99.3	90.1	97.5	98.4	85.5	94.4	97.5	77.5	88.9	43.4	72.8
−0.4	99.7	85.8	95.7	99.2	79.1	92.8	96.8	71.2	85.8	94.7	66.9	83.1	90.3	60.5	76.9		
0.0	99.0	67.7	86.1	95.8	60.2	80.7	92.3	57.2	76.6	85.6	51.4	69.9	81.6	46.4	66.0		
0.4	94.3	54.3	74.5	89.0	46.1	70.2	84.8	42.6	61.9	79.7	40.7	56.8	76.4	39.1	60.2		
0.8	88.2	42.7	66.7	81.4	37.8	59.5	77.7	37.2	58.2	76.3	35.7	57.7	68.9	31.4	48.8		
Admixture																	
−0.8	99.9	99.3	99.7	99.4	94.7	97.3	98.7	87.9	94.6	94.6	77.5	88.2	93.1	70.1	78.6	40.3	63.2
−0.4	98.2	83.4	96.2	94.9	70.2	90.7	90.8	64.1	83.1	86.8	58.9	78.4	81.0	54.7	74.3		
0.0	92.0	61.2	87.7	87.6	57.0	78.9	83.3	52.6	75.5	78.9	47.3	72.3	74.5	42.9	65.8		
0.4	82.7	45.8	72.1	78.8	42.6	69.9	73.2	40.8	63.4	68.7	39.0	59.6	64.1	35.0	57.2		
0.8	76.9	41.3	65.2	69.8	36.4	61.7	63.1	34.7	55.4	62.3	32.5	54.1	57.8	29.5	39.5		

Parents were deleted from the simulation. See Table 1 and 2 legends for simulation and abbreviation details.

We also study the effects of two factors, including the level of LD and family structure, on power estimations. As presented in table 6, all the methods have increased power with increased *r* ^2^. As the number of children per family increases, the power of the proposed method and UT decreases a little, whereas that of the other methods increases instead. Again, the proposed method has the highest power in all situations.

**Table 6:** The effects of LD level and family structures on power estimates.

	No. of children per family														
	1					2					3				
*r* ^2^	T	FBAT	PBAT	QTDT	UT	T	FBAT	PBAT	QTDT	UT	T	FBAT	PBAT	QTDT	UT
Homogeneous															
0.25	46.2	14.7	17.3	14.5	41.1	42.1	15.7	20.9	18.1	43.6	41.9	18.9	23.4	20.8	40.8
0.50	72.7	18.2	26.4	20.5	70.3	74.6	26.1	37.9	30.4	66.2	72.2	34.2	41.1	36.3	67.9
0.75	89.4	28.8	39.0	31.5	86.0	91.0	41.3	53.3	42.0	85.1	89.5	49.5	59.7	50.8	83.7
1.00	97.1	36.9	49.5	39.6	92.8	96.0	52.2	63.7	54.0	92.4	95.7	59.9	70.5	62.5	92.5
Admixture															
0.25	32.5	11.4	15.4	13.2	31.6	33.0	13.7	18.9	16.5	29.0	29.2	12.8	19.5	16.4	33.1
0.50	60.8	13.9	18.2	17.7	58.2	61.0	22.9	34.8	28.8	58.7	60.6	25.1	35.4	30.7	57.5
0.75	80.9	22.2	36.3	26.7	77.4	81.6	32.2	48.6	37.6	75.7	79.5	33.4	55.9	43.7	74.2
1.00	92.3	25.4	41.6	34.1	87.1	92.4	42.0	57.1	49.3	86.3	91.9	45.7	66.8	55.6	89.5

The number of children per family varied from 1 to 3, with the total number of individuals being constrained at 800. See Table 1 and 2 legends for simulation and abbreviation details.

### Analysis of GAW16 Datasets

**Figure 1:**
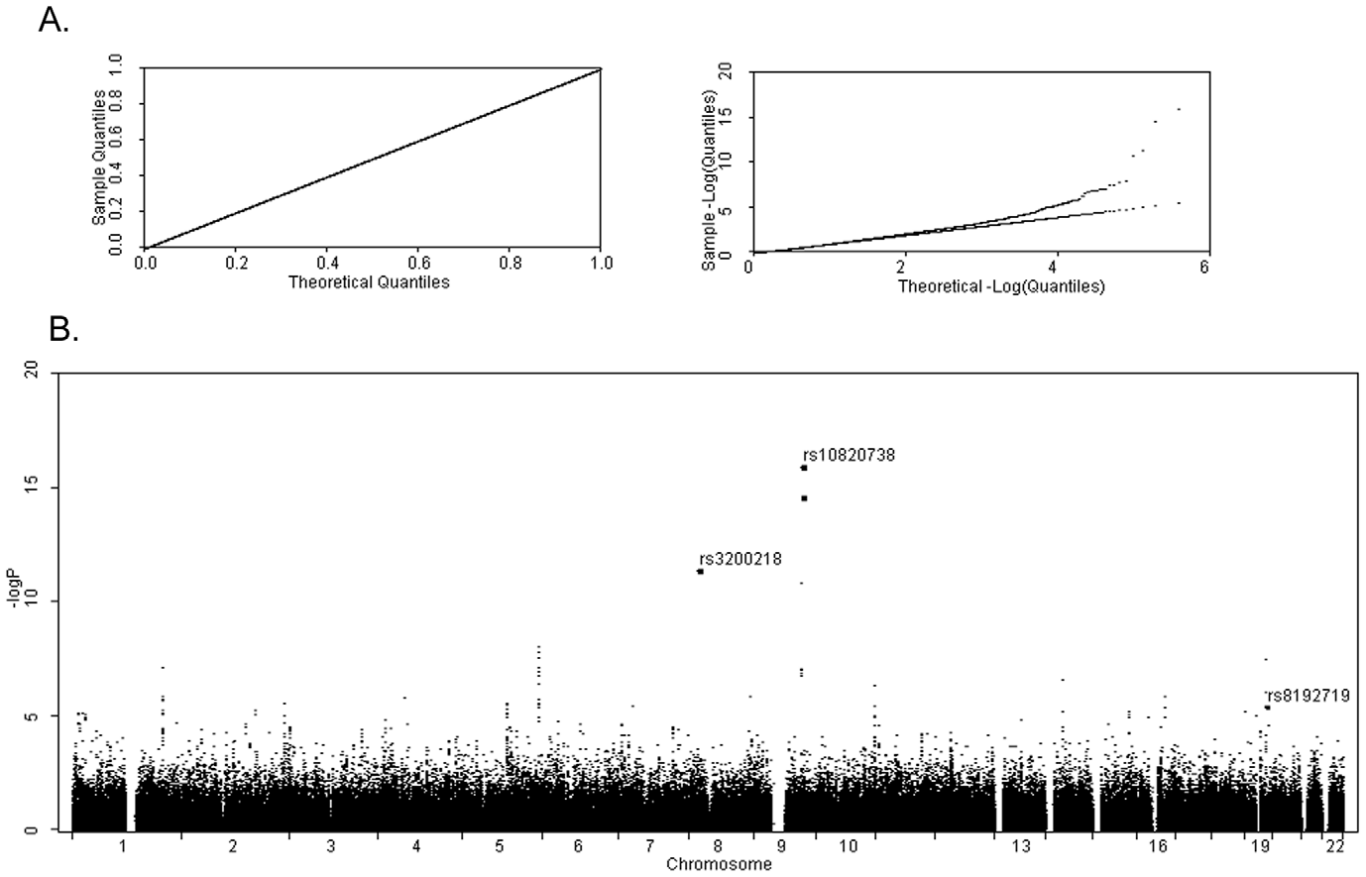
Application of the proposed method to the GAW16 simulated datasets. The GAW16 simulated HDL and TG traits were analyzed. Figure 1A, the quantile-quantile (QQ) plot (left), and log-QQ plot (right); Figure 1B, raw p-values of the genome-wide scan.

As an application, we analyze the GAW16 simulated datasets as described in the preceding section. On a desktop computer with a single 2.8GHz Intel Xeon CPU, the computation from the proposed method for the scan with 550K SNPs takes a total time of approximately 20 hours. Figure 1A presents the quantile-quantile (QQ) plot (left) and log-QQ plot (right), and Figure 1B plots raw p-values over 22 autosomes. Obviously, the overall p-values distribute uniformly between 0 and 1. The most significant signal from the proposed method is observed at SNP *rs10820738*, with a p-value 9.55e-17. UT has a p-value 4.42e-17 at this SNP. The second most significant signal corresponds to one of its flanking SNPs *rs2297398* (7.7kb apart), with a p-value 1.88e-15 from the proposed method. The SNP *rs10820738* explains the most phenotypic variation (1.0%) for HDL trait in the GAW16 simulation, but none for TG trait. The third most significant SNP is *rs3200218* with a p-value 3.47e-12. This SNP is exactly one of the two SNPs that present pleiotropic effects to both traits. The p-value at the other major pleiotropic SNP *rs8192719* is 3.07e-6, which does not achieve the genomewide significance level. However, one of its flanking SNPs *rs7249735* (12.8kb apart) presents a p-value 2.77e-8 that is significant at the genomewide level. Generally speaking, the proposed method successfully identifies both pleiotropic SNPs at the genomewide level. We also list p-values at these two SNPs from other methods in Table 7. Obviously, most of them do not achieve genomewide significant level, further demonstrating the advantage of the proposed method.

**Table 7:** P-values at pleiotropic SNPs.

	p-value				
SNP	T	FBAT	PBAT	QTDT	UT
*rs3200218*	3.47e-12	0.03	5.20E-04	1.91E-04	2.72e-08
*rs8192719*	3.07e-6	0.94	0.04	0.04	2.38e-06

For univariate tests, e.g., QTDT and UT, the uniform bivariate p-value was obtained by adjusting the minimum of the two univairate p-values by multiple testing correction (multiplying by 2). See Table 1 legend for abbreviation details.

## Discussion

In this study, we have presented a bivariate test of association for quantitative traits in families, by the use of the multivariate variance-components model. In particular, the proposed method uses principal component analysis to correct for population stratification. Simulation studies have shown that the proposed method not only outperforms the analysis focused on individual traits when pleiotropic effect is present, but also has increased power compared with the existing bivariate methods, while correcting for population stratification.

The strength of bivariate analyses is influenced by correlations between traits. Our simulation results show that bivariate analyses are more powerful when the major genes and the residual factors act in more dissimilar ways. For example, by constraining the major-gene correlation to +1.0, bivariate approaches are most powerful when both *ρ_a_* and *ρ_e_* are equal to −0.8, corresponding to an approximate phenotypic correlation of −0.76. When pleiotropic effect is present, bivariate analysis is more powerful than univariate analysis unless the residual correlation is high and in the same direction to the correlation of major gene effect, coinciding with the findings in linkage studies [27]. When pleiotropic effect is not present and there is weak or no correlation between traits, on the other hand, bivariate analysis is less powerful than univariate analysis [28]. In our analyses of the SNP *rs10820738* that has no pleiotropic effect in the GAW16 simulation, the p-value from the proposed bivariate method, 9.55e-17, is slightly higher than that from the univariate method UT, 4.42e-17. In practical applications in which the existence of pleiotropic effect is unknown *in prior*, bivariate analysis is not necessarily more powerful than univariate analysis, even when the traits are strongly correlated. Bivariate analysis should thus be processed with caution, and combing the results of bivariate and univariate analyses is warranted.

Thus, our simulations provide a statement in demonstrating that bivariate approaches are more powerful than univariate analyses unless major-gene effects and residual effects are very highly correlated in the same direction, which coincides with the conclusion of Amos et al. [27].

Our method that is developed by extending the variance-components model offers a way to powerfully/robustly perform bivariate association analysis in the presence of linkage in general pedigrees. The variance-components model is advantageous in detecting QTL in the following two aspects: first, it combines the analysis of linkage and association and would increase the power of detecting QTL when the marker, itself is not the QTL, is associated with the QTL. Second, the prior evidence on linkage can be incorporated to indicate LD strength between the QTL and the marker [29].

Another strengthen of our method is decomposing individual genotype scores by principal component analysis rather than by TDT-like strategy for controlling population stratification. The resulting test statistic provides largely improved power over existing TDT-based methods, where the latter may be prohibitive for application to genome scans due to their poor powers. For example, under the moderate setting where both polygenic and environmental correlation coefficients were set to 0.4 and the locus effect were set to 2%, we observed 164 and 24 significant results over 1,000 replicates for the proposed method and UT, respectively, but only 6, 8, and 4 for FBAT-GEE, FBAT-PC, and QTDT, respectively, at the genome-wide level 1.0e-6.

An interesting observation from our simulations is that family structures influence the power of the investigated methods in different patterns. For FBAT-GEE, FBAT-PC and QTDT that control population stratification through TDT, small numbers of large families provide more power than large numbers of small families. This is not surprising, since with these methods the parental information is used to control the stratification, and consequently, only the information of children contributes to test statistics. Contrary, the power decreases slightly as the number of children per family increases for the proposed method. This appears to be caused by the fact that a large number of small independent families provides more information on allele distributions than a small number of larger families can provide.

In this manuscript, we focus our attention on data with nuclear families. However, the proposed method is applicable to extended pedigrees as well. The described multivariate variance-components model can be directly applied to extended pedigrees. As for correcting for population stratification, the extension of principal component analysis coupled with TDT strategy to extended pedigrees is also straightforward. For example, all founders can be collected to form an unrelated sample. In cases where there is no founder, one sib can be randomly selected into the unrelated sample, as we described in reference [18]. PCA will then be performed on the unrelated sample, and both the genotype and the phenotype for each subject in the unrelated sample are adjusted accordingly. For subjects not in the unrelated sample, their principal components can be calculated by that of their parents or sib that is in the unrelated sample. The process will carry on recursively until all non-founders are adjusted. Some specialized algorithms, e.g., the one described in [30], can be adopted in a relatively simple manner. However, the performance when applying to extended pedigrees is unknown deserves further endeavor.

In summary, we develop a novel method for family based bivariate association test. Our method is more powerful than currently available bivariate methods. The proposed method is computationally effective and can complete a typical GWAS scan within hours. The program implementing our proposed method is available upon request to us.
